# Role of deep learning methods in screening for subcutaneous implantable cardioverter defibrillator in heart failure

**DOI:** 10.1111/anec.13028

**Published:** 2022-12-16

**Authors:** Mohamed ElRefai, Mohamed Abouelasaad, Benedict M. Wiles, Anthony J. Dunn, Stefano Coniglio, Alain B. Zemkoho, John M. Morgan, Paul R. Roberts

**Affiliations:** ^1^ Cardiac Rhythm Management Research Department University Hospital Southampton NHS Foundation Trust Southampton UK; ^2^ Faculty of Medicine University of Southampton Southampton UK; ^3^ Aberdeen Royal Infirmary NHS trust Aberdeen UK; ^4^ School of Mathematical Sciences University of Southampton Southampton UK

**Keywords:** artificial intelligence, heart failure, machine learning, subcutaneous implantable cardiac defibrillator, sudden cardiac death

## Abstract

**Introduction:**

S‐ICD eligibility is assessed at pre‐implant screening where surface ECG traces are used as surrogates for S‐ICD vectors. In heart failure (HF) patients undergoing diuresis, electrolytes and fluid shifts can cause changes in R and T waves. Subsequently, T:R ratio, a major predictor of S‐ICD eligibility, can be dynamic.

**Methods:**

This is a prospective study of patients with structurally normal hearts and HF patients undergoing diuresis. All patients were fitted with Holters® to record their S‐ICD vectors. Our deep learning model was used to analyze the T:R ratios across the recordings. Welch two sample *t*‐test and Mann–Whitney U were used to compare the data between the two groups.

**Results:**

Twenty‐one patients (age 58.43 ± 18.92, 62% male, 14 HF, 7 normal hearts) were enrolled. There was a significant difference in the T:R ratios between both groups. Mean T: R was higher in the HF group (0.18 ± 0.08 vs 0.10 ± 0.05, *p* < .001). Standard deviation of T: R was also higher in the HF group (0.09 ± 0.05 vs 0.07 ± 0.04, *p* = .024). There was no difference between leads within the same group.

**Conclusions:**

T:R ratio, a main determinant for S‐ICD eligibility, is higher and has more tendency to fluctuate in HF patients undergoing diuresis. We hypothesize that our novel neural network model could be used to select HF patients eligible for S‐ICD by better characterization of T:R ratio reducing the risk of T‐wave over‐sensing (TWO) and inappropriate shocks. Further work is required to consolidate our findings before applying to clinical practice.

## Graphical abstract

S‐ICD eligibility has the tendency to fluctuate overtime, particularly in patients with heart failure. Deep learning methods could enable more accurate and efficient screenings and the adoption of novel mathematical approaches for data analysis to determine HF patient eligibility for S‐ICD therapy seems promising.

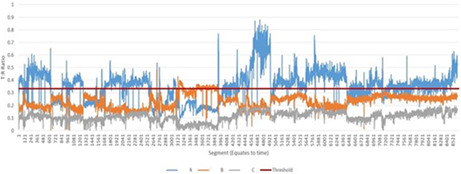

## 1 INTRODUCTION

We report a novel application of artificial intelligence and deep learning methods used to screen patients for S‐ICD eligibility. Screening data are acquired over a much longer period than for conventional screening approaches and provide an in‐depth description of the behavior of the T:R ratio over that period across the three S‐ICD vectors (Dunn et al., 2021). We hypothesize that this novel screening approach could enable more reliable and descriptive screening to better assess patient eligibility for S‐ICD implantation with lower risk of inappropriate shock therapy.

## 2 METHODS

**FIGURE 1:**
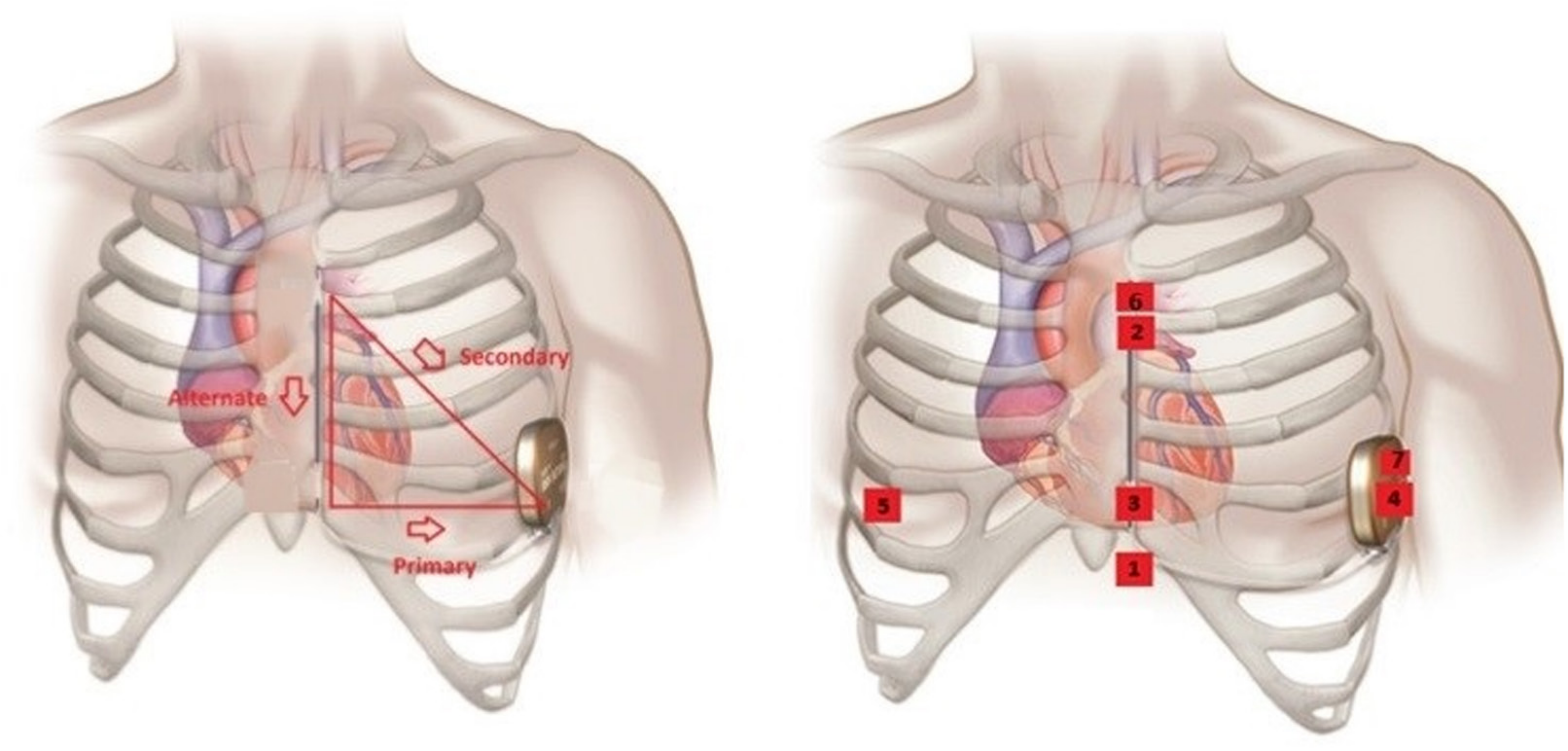
Showing the typical S‐ICD vectors on the left and on the right, the Holter® surface ECG positions. 1 = 1 cm infero‐lateral to the xiphisternum. 2 = 14 cm superior to position 1. 3 = 5th intercostal space, parasternal position. 4 = 6th intercostal space left mid axillary line. 6 = Adjacent to 2. 7 = Adjacent to 4. Holter Channel A records between points 1 and 4 = surrogate of S‐ICD primary vector. Holter Channel B records between points 2 and 3 = surrogate of S‐ICD alternate vector. Holter Channel C records between points 6 and 7 = surrogate of S‐ICD secondary vector. 5 = 5th intercostal space right midclavicular line = neutral electrode. Image prior to annotation © Boston Scientific Corporation or its affiliates.

This is a prospective observational study on healthy volunteers with structurally normal hearts and patients with a known history of heart failure admitted for diuresis on clinical grounds. None of the recruited patients had an ICD (TV‐ICD or S‐ICD). All the participants were asked to wear a seven lead/three channel Holter® monitors for 24 h. The leads for the Holters® were positioned so that they mimic and correspond to the three vectors (primary, alternate, and secondary) of an S‐ICD (Figure 1).

The aim of our study was to quantify, describe and compare the degree of variation in T:R ratio observed in patients with HF and healthy participants with structurally normal hearts from an S‐ICD vector perspective.

T:R ratio was chosen specifically as the parameter to be tracked and analyzed by our novel tool because of the crucial role of the T:R ratio in the sensing mechanism of the S‐ICD and its subsequent determination of S‐ICD eligibility and TWO events. A T:R ratio eligibility cut‐off ratio of 1:3 was chosen based on the manual S‐ICD screening tool following the manufacturer's guidelines (Randles et al., 2014), although the manual screening method is now highly replaced with automatic screening methods, alas they follow the same principles.

Patients were recruited to the HF group based on clinical diagnosis of HF regardless of underlying left ventricular function on echocardiography and having received intravenous diuretic therapy (at least 120 mg furosemide/24 h) on clinical grounds under the discretion of the treating physician. Patients' demographics (age, gender, cardiovascular history, LV function) were obtained from the medical records. There was no requirement for further patient follow‐up. The study was performed REC (17/SC/0623) approval and was granted R&D (RHMCAR0528) approval.

Raw data from the Holters were downloaded in ASCII (American Standard Code for Information Interchange) format at a frequency of 500 Hertz (Hz). A bespoke tool developed by Dunn et al efficiently and accurately tracked and analyzed the T:R ratio for the leads corresponding to the S‐ICD vectors over the 24‐h recordings period (Dunn et al., 2021).

## 3 ARTIFICIAL INTELLIGENCE AND NEURAL NETWORKING MODEL

Machine learning methods are already being used in a variety of applications such as the classification and the prediction of various cardiovascular diseases through ECG data analysis (Fan et al., 2018; Kiranyaz et al., 2016; Lih et al., 2020; Pourbabaee et al., 2018; Roberts et al., 2001; Rocha et al., 2008; Vemishetty et al., 2016; Vemishetty et al., 2019; Zhang et al., 2020). A well‐recognized technique for preprocessing ECG data is to create its phase space reconstruction matrix (PSR). Typically, manually selected features such as box counting as well as column and row statistics are extracted from the PSR of the ECG data which then can be used as inputs for a classification model. Convolutional neural networks (CNNs) have been used in ECG analysis for classifying heart attacks, atrial fibrillation, and other arrhythmias as well as for predicting blood pressure (Cho et al., 2020; Fan et al., 2018; Jo et al., 2021; Lih et al., 2020; Liu et al., 2018; Miao et al., 2020; Pourbabaee et al., 2018; Sangaiah et al., 2020; Zhang et al., 2020; Zhu et al., 2020).

The method we proposed diverges from standard approaches by using the whole PSR matrix as the input to a CNN model which to the best of our knowledge has not been attempted before. The proposed method is capable of automatically extracting a set of features that are much more descriptive than those that are found manually with more time‐consuming methods.

For our tool, the data (in ASCII format) were first split into 10 s segments. Baseline drift correction techniques were applied, followed by adaptive band stop filtering to suppress power‐line noise with a frequency of 50 Hz while a low pass filter was used to remove the remaining high‐frequency noise. Then, PSR—a popular technique in waveform analysis for representing non‐linear characteristics of time series set of data using delay maps—was utilized to convert the ECG signal into a compressed 32 x 32 pixel PSR image, one image for each 10 s worth of ECG data. A Convolutional Neural Network (CNN) model was then trained to predict the T:R ratio from these PSR images with a high degree of accuracy without explicitly locating the R or T waves. The end result is a plot showing the variation of the T:R ratios for each lead/S‐ICD vector over the recorded period (24 h in our study), where, for readability, the line graph is smoothed to where each point gives the average T:R ratio for the preceding half hour, thus making it easy to detect any period where the T:R ratio was consistently high and thus increased the risk of TWO. To better examine how the behavior of the T:R ratio differs between each lead, our tool can plot a histogram of what proportion of the 24‐h screening period the T:R ratio of a particular lead spent in each range of T:R ratios (Dunn et al., 2021).

We note that it is more standard in the literature to consider the R:T ratio as opposed to the T:R ratio. Despite this, as the T‐wave amplitude approaches 0, subtle changes in the T‐wave amplitude can result in extreme variations in the R:T ratio, which makes the latter inappropriate for use as a label in our model. For this reason, we use the T:R ratio as a dependent variable in our regression problem.

### 3.1 Statistical methodology

The distribution of the data was identified using histograms, QQ plots, and box plots. Parametric data were described using mean ± standard deviation (SD), and categorical data as *n*/*N* (%). The Welch two sample *t*‐test, Wilcoxon rank test and Mann–Whitney U were used to compare between the continuous variables in the two groups.

## 4 RESULTS

Twenty‐one patients were recruited into two groups: 7 patients in the structurally normal heart group and 14 patients in the heart failure group. The mean age was 58.43 ± 18.92 years (62% male) (Table 1). Age and gender were not significantly different for either mean or the standard deviation (SD) of the T:R ratio.

**TABLE 1:** Patients' demographics

Total number of participants		*N* = 21		Heart failure *N* = 14	Structurally normal heart *N* = 7
Demographics:	Mean age [years ± 95% CI]	58.43 ± 18.92		70 ± 11	36 ± 8
	Male	13	61.9%	10/14 (71%)	3/7 (43%)
Cardiac co‐morbidities:	Heart failure	14	66.67%	14	0
	Atrial fibrillation	6	28.57%	6 (42.85%)	0
	LV diastolic dysfunction	4	19.05%	4 (28.57%)	0
	Ischemic heart disease	6	28.57%	6 (42.85%)	0
	LV systolic dysfunction	10	47.62%	10 (71.43%) Ejection fraction % = 25.3 ± 6.97 [95% Cl]	0
Fluid loss in 24 h in mls for the HF group				2326.07 ± 1253.18 [95% Cl]	
Furosemide dose in 24 h in mgs for the HF group				257.86 ± 45.86 [95% Cl]	
Shift in Na levels before and after diuresis [mmol/l] for the HF group				1.93 ± 0.73 [95% Cl]	
Shift in K levels before and after diuresis [mmol/l] for the HF group				0.49 ± 0.27 [0.95 Cl]	

**FIGURE 2:**
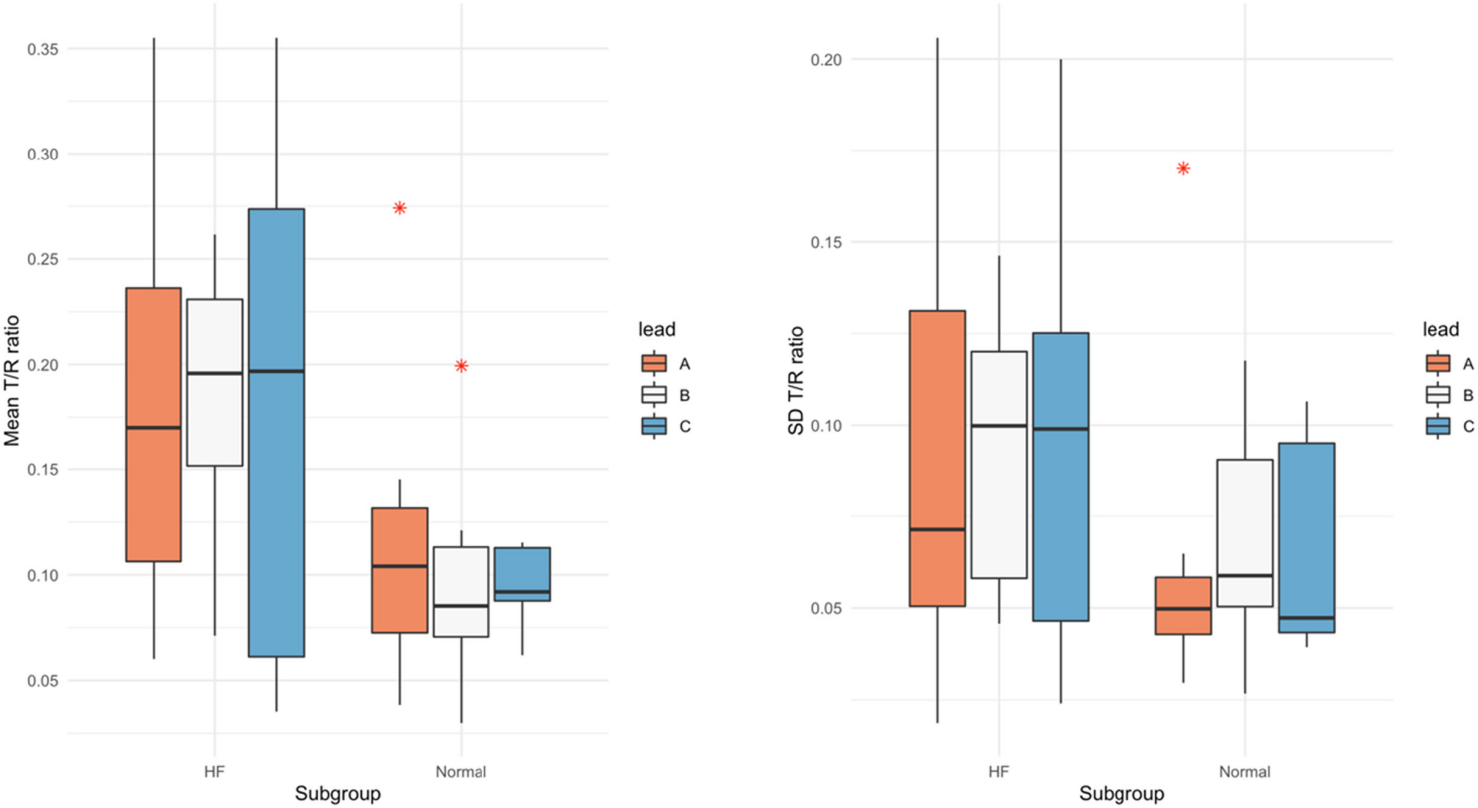
Box plots for the mean (*p* < .001) and standard deviation (*p* = .02) of the T:R ratio over 24 h screening period in the studied subgroups (heart failure patients undergoing diuresis vs healthy volunteers with structurally normal hearts). Leads A, B and C correspond to primary, alternate, and secondary vectors of an S‐ICD respectively.

Mean T:R ratio was higher in the HF group (0.181 ± 0.084 vs 0.104 ± 0.054, *p* < .001), and the SD (a measure of dynamicity) of the T:R ratio was also higher in the HF group (0.093 ± 0.048 vs 0.067 ± 0.036, *p* = .02). There was no significant difference found in the mean or the SD of the T:R ratio between different leads within the same group (Table 2, Figure 2).

**TABLE 2:** Comparison between the parameters of the T: R between both groups

Parameter	Group		*p* value
	Heart failure	Structurally normal heart	
Mean T:R ratio	0.181 ± 0.084 (95% CI)	0.104 ± 0.054 (95% CI)	<.001 (Welch two sample *t*‐test)
Standard deviation of T:R ratio	0.093 ± 0.048 (95% CI)	0.067 ± 0.036 (95%CI)	=.024 (Welch two sample *t*‐test

To highlight the impact of the differences in the mean and SD of T: R between both groups, the percentage of time when the T:R ratio was found to be above the screening threshold (1/3) for the T:R ratio (unfavorable) was calculated and compared in both groups. The heart failure group had an unfavorable T:R ratio for significantly longer time in the 24‐h tape compared to the normal group, this was also evident in all the vectors: (13.3% ± 10% vs 4% ± 8%, *p* = .26) in the primary vector, (9.7% ± 7.4% vs 3% ± 4%, *p* = .29) in the alternate vector, and (10.8% ± 12.8% vs <1%, *p* = .22) in the secondary vector (Table 3).

**TABLE 3:** Differences in the “unfavorable” T:R ratios in both groups

ID	Group	Primary vector		Alternate vector		Secondary vector	
		10‐s segments of T: R > 1/3 *N* = 8640	Proportion of the 24‐h recording (%)	10‐s segments >1/3 *N* = 8640	Proportion of the 24‐h recording (%)	10‐s segments >1/3 *N* = 8640	Proportion of the 24‐h recording (%)
1	Normal	3	<1%	0	0%	23	<1%
2	Normal	2613	30%	19	<1%	0	0%
3	Normal	17	<1%	51	<1%	0	0%
4	Normal	1	<1%	3	<1%	1	<1%
5	Normal	0	0%	452	5%	14	<1%
6	Normal	2	<1%	12	<1%	277	3%
7	Normal	1	<1%	1074	12%	3	<1%
	Mean	377 ± 731	4% ± 8%	230 ± 301	3% ± 4%	45 ± 76	<1%
8	HF	8	<1%	1524	17.6%	NA	NA
9	HF	1024	11.9%	815	9.4%	NA	NA
10	HF	1280	14.8%	219	2.5%	NA	NA
11	HF	0	0%	1828	21.2%	NA	NA
12	HF	2305	26.7%	637	7.4%	NA	NA
13	HF	0	0%	31	<1%	NA	NA
14	HF	1104	12.8%	5	<1%	37	<1%
15	HF	3555	41.1%	4652	53.8%	4054	46.9%
16	HF	389	4.5%	452	5.2%	291	3.4%
17	HF	5562	64.4%	417	4.8%	0	0%
18	HF	13	<1%	400	4.6%	222	2.6%
19	HF	808	9.4%	383	4.4%	2866	33.2%
20	HF	0	0	0	0	0	0
21	HF	2	<1%	406	4.7%	1	<1%
	Mean	1146 ± 862	13.3% ± 10%	841 ± 640	9.7% ± 7.4%	934 ± 1105	10.8% ± 12.8%

To summarize the results, T:R ratios of S‐ICD vectors were higher at the baseline and exhibited more fluctuations in the HF group. This has translated into higher likelihood of unfavorable crossing of the screening threshold in HF patients when compared to the structurally normal heart group.

## 5 DISCUSSION

Heart failure (HF) is a global cardiovascular disease with an estimated prevalence of more than 37.7 million patients worldwide affecting 1%–2% of adults in developed countries (Chaudhry & Stewart, 2016). A high proportion of deaths among patients with HF occur suddenly and can be attributed to ventricular arrhythmias. As such many international guidelines recommend using implantable ICDs to reduce sudden death in patients with heart failure. Thus an ICD is recommended to reduce the risk of sudden death and all‐cause mortality in patients with symptomatic HF (NYHA Class II–III), and an LVEF ≤35% despite ≥3 months of optimal medical therapy (Class IA and IB indications in patients with ischemic heart disease and patients with dilated cardiomyopathy respectively Ponikowski & Voors, 2017).

Transvenous ICDs employ transvenous (intracardiac) leads for rhythm discrimination and delivery of defibrillation shock therapy, and as such are associated with potential complications related to invasion of the vascular space. These comprise complications that can occur at the time of implants such as pneumothorax and cardiac tamponade due to traumatic placement of lead(s), and long‐term complications such as device infection of the device progressing to systemic sepsis and/or infective endocarditis with potentially fatal consequences. Additionally, ICD leads that remain in the vasculature for many years may, ultimately, compromise flow or cause obstruction.

The S‐ICD offers an alternative solution to the traditional TV‐ICD for prevention of sudden cardiac death in patients with heart failure. Studies have confirmed comparable efficacy to TV‐ICD but the S‐ICD avoids many of the complications associated with TV‐ICDs and may be the only ICD therapy for patients with no venous access or at high risk of infective endocarditis (Kamp & Al‐Khatib, 2019). Thus the AHA/ACC/HRS Guidelines for Ventricular Arrhythmia and Sudden cardiac death have a Class I recommendation for S ‐ICD implantation in patients who are at a high risk for infection, or have no appropriate venous access and who have no indication for bradycardia or biventricular pacing and/or anti‐tachycardia pacing (ATP) (Kamp & Al‐Khatib, 2019).

However, not all patients are eligible for an S‐ICD. Eligibility is identified during a recommended pre‐implant screening process that is undertaken in all potential recipients. An S‐ICD programmer with a built‐in automated screening tool is utilized in screening. It has external ECG cables which can acquire ECG traces via positioning the skin electrodes on the chest wall using the same anatomical landmarks that would guide future S‐ICD implantation. As such, obtained ECG traces—usually of short duration of few seconds in multiple postures—act as a surrogate for the three S‐ICD vectors allowing non‐invasive assessment of vector morphology and S‐ICD eligibility. A major predictor of eligibility of a vector is the T:R ratio which is unique for every vector as varying the angle of recording alters the amplitude of both R wave and T wave. Vectors with lower T:R ratios are more likely to pass the screening and are safe for clinical use, while a vector that fails cannot be used in clinical practice. To be eligible for an S‐ICD a patient requires at least a single vector to pass screening in at least two postural positions at the same amplitude. Patients with vectors that do not meet the screening criteria are at high risk of TWO and deemed ineligible for an S‐ICD. This is important as inappropriate shock therapies can have detrimental effects on the quality of life, psychological well‐being and can even result in the induction of ventricular arrhythmias (Daubert et al., 2008). Despite the current screening process, the incidence of inappropriate shocks is greater in S‐ICDs when compared with conventional TV‐ICDs and the most common reason for inappropriate shocks in S‐ICDs is T‐wave oversensing (Boersma et al., 2017).

It is important to note that temporal variations in R wave and T wave amplitudes in the same individual are frequently observed on ECG recordings and thus, the T:R ratio—a major predictor of S‐ICD eligibility—is not fixed in any given individual. Factors such as changes in posture and heart rate can influence ECG parameters. Also changes in electrolytes concentrations, body weight, fluid shifts, and lung congestion can cause detectable dynamic changes on surface ECG recordings (Al‐Zaiti et al., 2011; Assanelli et al., 2013; Hasan et al., 2012; Madias, 2005; Madias et al., 2001; Walker et al., 2003). HF patients share a lot of the factors that cause variation in the ECG components, particularly patients with significant changes in their weight and shifting of their body fluid status over short time such as heart failure patients undergoing diuresis. The mere presence of LV dysfunction is an independent factor contributing to the variation of ECG parameters over time (Fosbøl et al., 2008).

Rhythm discrimination by the S‐ICD and its vector sensing algorithms has been shown to be non‐inferior to TV‐ICD systems (Gold et al., 2012). However, it is important to ensure that the S‐ICD system does not “over sense” T waves which can lead to inappropriate arrhythmia detection and shock therapy. This occurs when the T wave is of greater amplitude than the sensitivity level of the device and is miscounted as an R wave such that the device misinterprets a single heartbeat (QRS complex followed by a T wave) as two separate R waves with a short R: R interval, so doubling the detected heart rate.

We identified these signal analysis processes for S‐ICD screening in HF patients as being suited to a novel mathematical approach employing artificial intelligence and neural networks analyzing vector data recorded over a 24‐h period.

**FIGURE 3:**
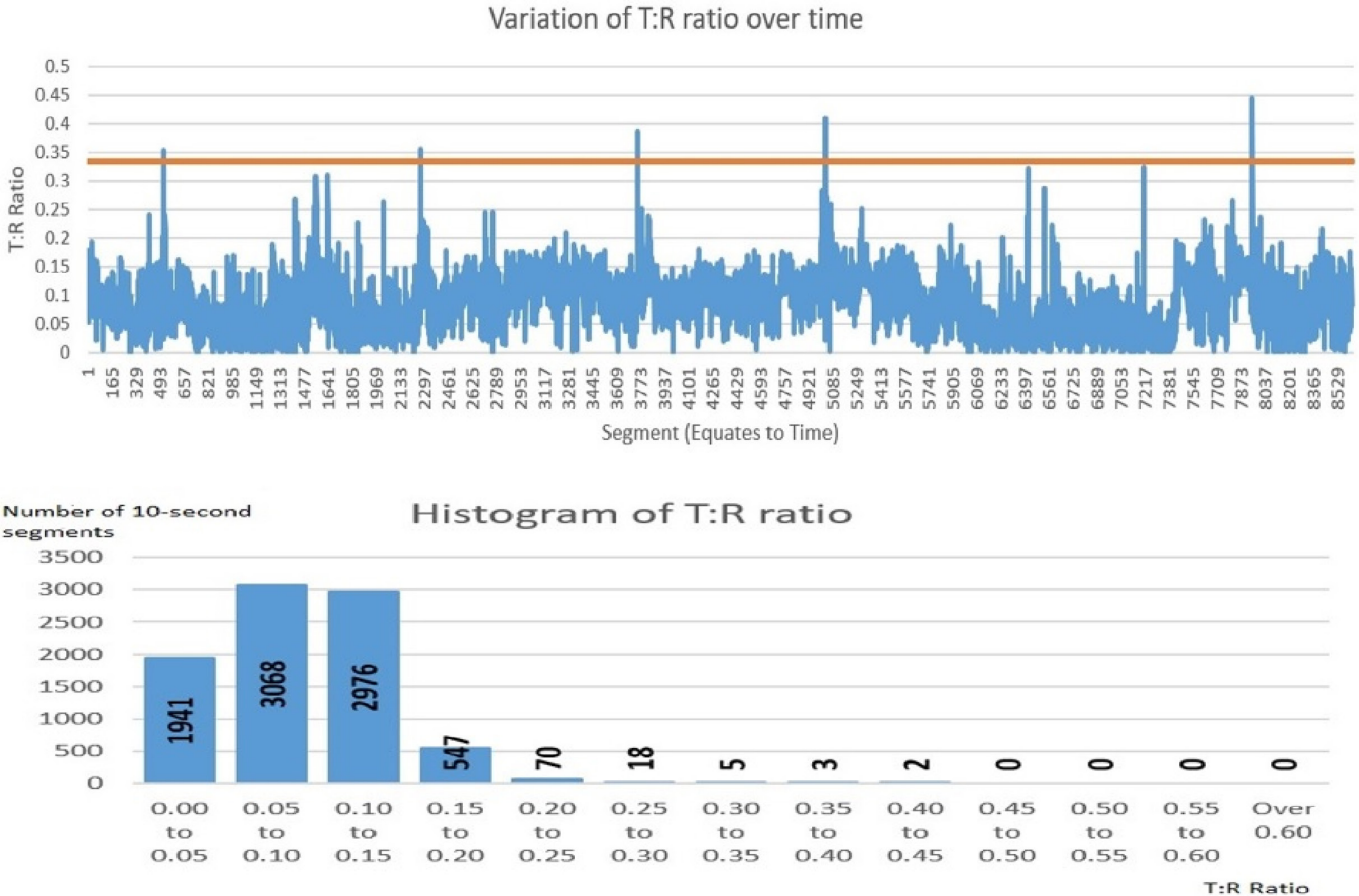
An example of the T:R ratio fluctuating overtime crossing the S‐ICD screening threshold for the T:R ratio (0.33) on multiple occasions over the 24‐h period. The histogram illustrates the exact number of 10‐s segments at each T:R ratio throughout the 24‐h recording. The above example represents the alternate vector for one of the patients who was recruited to the HF group.

The concept of the potential varying of S‐ICD vectors eligibility over time was previously presented in a study by (Wiles et al., 2021). The study demonstrated that the vector score which determines S‐ICD eligibility is in fact dynamic in real‐life ICD population. Our approach demonstrated that one of the main determinants of S‐ICD eligibility—the T:R ratio—is in fact dynamic. The changes in the T:R ratio in some of the vectors that were observed over time in our cohort of patients were significant enough in some instances to cause the T:R ratio to cross the threshold for the S‐ICD screening (Figure 3).

The T:R ratios were unfavorable a priori in the HF group when compared to the normal hearts group. Importantly T:R ratios were more likely to fluctuate and cross the S‐ICD screening threshold in HF patients than in the normal heart patients. The cohort of HF patients in our study shared many characteristics, such as rapid fluid and body weight shifts and quick changes in electrolyte concentrations, known to cause dynamic changes in ECG signals.

**FIGURE 4:**
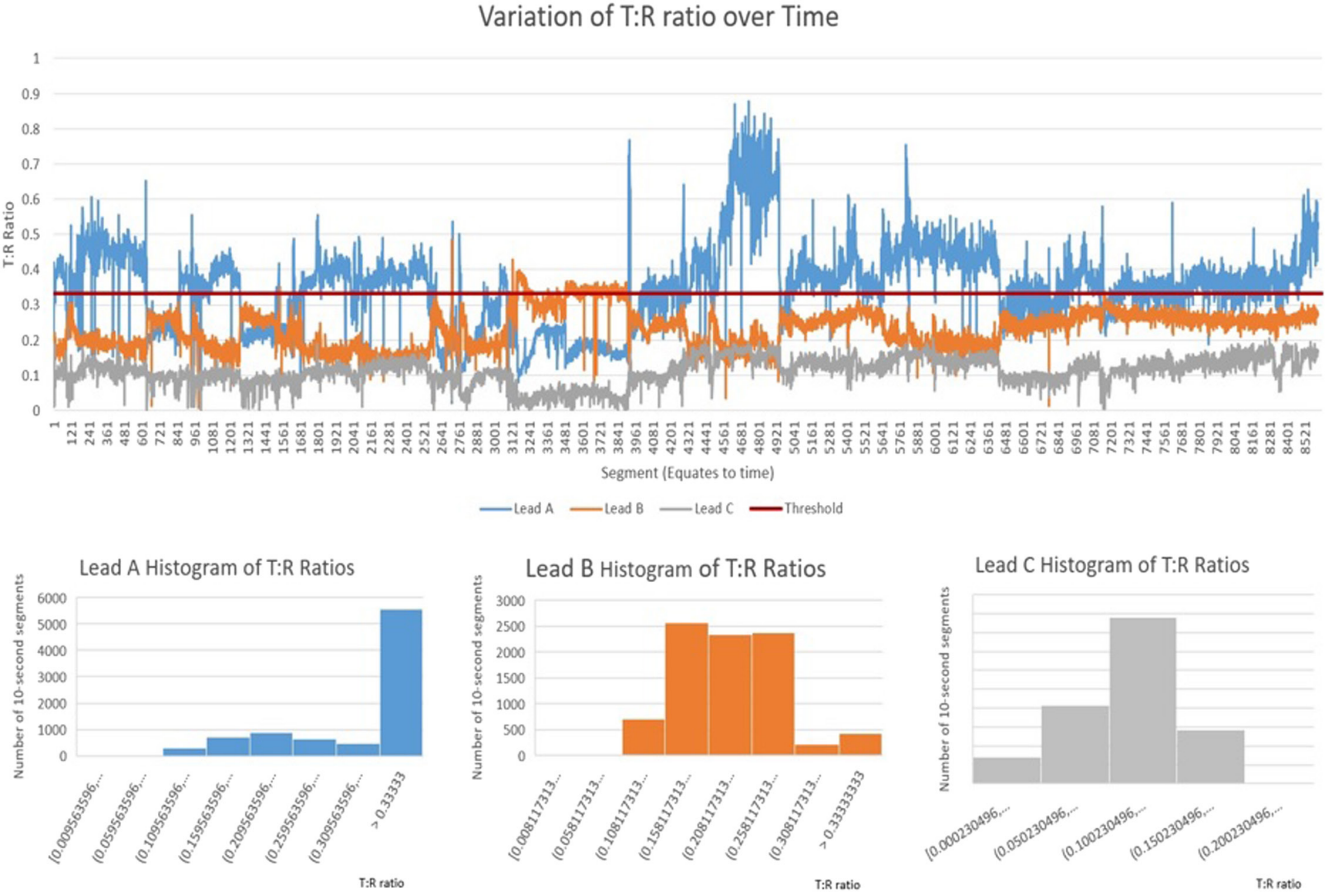
An example of how our tool can help select the most suitable vector for programming the S‐ICD. The analysis of the Holter recording for one of the patient recruited in the HF group: All the 3 leads had acceptable T:R ratios at some stage of the 24‐h recordings, however, while the T:R ratios for leads A and B (corresponding to the primary and alternate vectors) showed significant fluctuations over the 24‐h recording and crossed the screening threshold multiple times, T:R ratio for Lead C (secondary vector) was stable in comparison and did not cross the threshold throughout the 24‐h posing the least risk of TWO.

In the event of multiple vectors passing the S‐ICD screening—not an uncommon occurrence—our tool can also guide the selection of the most favorable vector for programming the S‐ICD. The most favorable vector would be the most stable or the least likely to fluctuate and cross the screening threshold and thus theoretically pose the least risk of TWO and inappropriate shocks (Figure 4).

It is important to interpret our results with caution. Firstly, because of the relatively small number of patients involved in our study—though each patient provided significant amount of data on the behavior of the T:R ratio for the 3 standard S‐ICD vectors for a much longer duration than that currently used in the day‐to‐day practice. It is also important to note that none of the patients recruited to our study in either group had S‐ICD implants or were candidates for an S‐ICD. While it could be argued that our analysis will not apply to real‐life S‐ICD patients, many such S‐ICD recipients fall into either of our recruited patients' cohorts. Also, T:R ratio, despite being a key component in the S‐ICD sensing process, is not the only parameter and other factors that play a role in the S‐ICD sensing process such as QRS duration as well as the impact of the relatively newer S‐ICD sensing algorithms, i.e., Smart Pass, were not examined in our analysis. It is also important to note that—while theoretically relevant—there is no evidence that the fluctuations in the T:R ratios that were demonstrated in this study would inevitably lead to adverse clinical outcomes such as TWO and inappropriate shocks and further work is needed to appreciate the clinical significance of our findings.

## 6 CONCLUSIONS

T:R ratio, one of the integral components of the S‐ICD sensing mechanism and a main determinant of S‐ICD eligibility, has the tendency to significantly fluctuate overtime, particularly in patients with heart failure when compared to patients with structurally normal hearts. This poses a theoretical risk for TWO and inappropriate shocks in HF patients who have S‐ICDS fitted in after being found S‐ICD eligible following the current screening practices. Incorporating deep learning methods could enable more accurate and efficient screenings and the adoption of novel mathematical approaches for data analysis of longer, data‐rich, screening practices to determine HF patient eligibility for S‐ICD implantation seems promising. The principles of our study need to be tested in a larger, more diverse patient cohort and the clinical relevance of our findings needs to be further investigated before it is possible to apply our tool to clinical practice.

## 7 AUTHOR CONTRIBUTIONS

M. ElRefai involved in conceptualization, writing, data curation, analysis, and review and editing. M. Abouelasaad involved in data curation and analysis. B. Wiles involved in conceptualization, and review and editing. A. Dunn, S. Coniglio, and A. Zemkoho involved in data analysis and review. J. Morgan involved in review and editing. P. Roberts involved in conceptualization, data analysis, and review and editing.

## CONFLICT OF INTEREST

Mohamed ElRefai is receiving unrestricted grant from Boston Scientific. Ben Wiles has received unrestricted research funding and consultancy fees from Boston Scientific. Paul Roberts receives consultancy fees from Boston Scientific. John Morgan is a senior medical director, Rhythm Management at Boston Scientific. Other authors in the study has no financial disclosures to declare.

## ETHICAL APPROVAL

The study was performed REC (17/SC/0623) approval and was granted R&D (RHMCAR0528) approval. All patients enrolled in this study gave informed written consent to this study prior to participation.

## Data Availability

The data that support the findings of this study are available from the corresponding author upon reasonable request.
